# Transovarial transmission of *tomato yellow leaf curl virus* by seven species of the *Bemisia tabaci* complex indigenous to China: Not all whiteflies are the same

**DOI:** 10.1016/j.virol.2019.03.009

**Published:** 2019-05

**Authors:** Qi Guo, Yan-Ni Shu, Chao Liu, Yao Chi, Yin-Quan Liu, Xiao-Wei Wang

**Affiliations:** Ministry of Agriculture Key Lab of Molecular Biology of Crop Pathogens and Insects, Institute of Insect Sciences, Zhejiang University, Hangzhou 310058, China

**Keywords:** TYLCV, Vertical transmission, Whitefly, Indigenous species

## Abstract

Begomoviruses contain some of the most damaging viral disease agents of crops worldwide, and are transmitted by whiteflies of the *Bemisia tabaci* species complex. During the last 20 years, transovarial transmission of tomato yellow leaf curl virus (TYLCV) has been reported in two invasive species of the *B. tabaci* complex. To further decipher the importance of this mode of transmission, we analyzed transovarial transmission of TYLCV by seven whitefly species indigenous to China. TYLCV virions were detected in eggs of all species except one, and in nymphs of two species, but in none of the ensuing adults of all seven species. Our results suggest that these indigenous whiteflies are unable to transmit TYLCV, a begomovirus alien to China, via ova to produce future generations of viruliferous adults, although most of the species exhibit varying ability to carry over the virus to the eggs/nymphs of their offspring via transovarial transmission.

## 1 Introduction

In the past decades begomoviruses have emerged as important insect-borne plant viruses of agricultural significance (Navas-Castillo et al., 2011). Among the plant viruses currently recognized by the International Committee on Taxonomy of Viruses (ICTV), *Begomovirus* is the largest genus and some viruses of this genus have become serious constraints to the production of many crops in the tropics, subtropics and warm temperate regions (Navas-Castillo et al., 2011, Zerbini et al., 2017). Begomoviruses are circular plant DNA viruses characterized by geminate particles comprised of two joined incomplete icosahedra encapsulating single-stranded DNA genome molecules of about 2700 nucleotides (Zerbini et al., 2017, Hesketh et al., 2018). Tomato yellow leaf curl virus (TYLCV) is a whitefly vectored begomovirus and the causative agent of tomato yellow leaf curl disease, a severe plant disease affecting tomato production (Scholthof et al., 2011).

The whitefly *Bemisia tabaci* (Gennadius) (Hemiptera: Aleyrodidae) is considered a species complex containing over 35 putative cryptic species (here after “species”; De Barro et al., 2011; Liu et al., 2012; Boykin and De Barro, 2014). Whiteflies of this species complex cause damage to plants through direct feeding and transmission of plant viruses (Wang et al., 20, Walker et al., 2010, Luan et al., 2014). Two species in the complex, Middle East-Asia Minor 1 (MEAM1) and Mediterranean (MED), previously known as the B and Q “biotypes”, have caused serious damage to agriculture worldwide due to their rapid and widespread invasions around the globe in the past three decades (De Barro et al., 2011, Naranjo et al., 2010). Based on mitochondrial cytochrome oxidase I (mt*COI*), 19 species of the *B. tabaci* complex have been recorded in China, including 17 indigenous species and two invasive species MEAM1 and MED (Hu et al., 2011, Hu et al., 2018, Liu et al., 2012). The indigenous *B. tabaci* fauna is distributed in the southern, southeastern and southwestern regions of China (Hu et al., 2011).

Transmission of begomoviruses is affected by many biotic and abiotic factors (Czosnek et al., 2017, Wang et al., 2016). Previous studies have shown that different species of the *B. tabaci* complex may transmit a given begomovirus with different levels of efficiency, and each of the whitefly species may only be able to transmit certain viruses (Wei et al., 2015, Guo et al., 2015, Zhao et al., 2019). Case studies have shown that differential efficiency of a begomovirus to cross the midgut of different species of whiteflies results in variation of virus transmission by the vectors, and specific cells in the primary salivary glands of the whitefly control retention and transmission of begomoviruses (Pan et al., 2018a, Pan et al., 2018b, Wei et al., 2014). Further studies have shown that the interactions between whitefly proteins and begomovirus proteins could affect the acquisition and transmission of begomoviruses, and the endosymbionts in whitefly or the GroEL protein produced by them could facilitate viral transmission (Shalev et al., 2016, Pan et al., 2017, Czosnek et al., 2017, Kilot et al., 2014, Gottlieb et al., 2010, Xia et al., 2018). However, research on whitefly transmission of begomoviruses has mainly focused on the maintenance and spread of begomoviruses through plant-insect-plant cycle (Gray et al., 2014). Other routes of transmission, especially transovarial transmission, have attracted limited attention.

Transovarial transmission of viral nucleic acid in insect vectors has important epidemiological relevance, as the vector harbours a source of inoculum in the absence of host plants of the virus and can facilitate viral spread over long distances (Accotto and Sardo, 2010). In the period of 1960–1980, when many new viruses were discovered or characterized, interest in vertical transmission began to attract attention from researchers of geminiviruses (Czosnek et al., 2017). Butter and Rataul (1977) could not demonstrate transovarial transmission of tomato leaf curl virus (TLCV) but the species and the developmental stage of the whitefly used in the test were not mentioned. Working with TYLCV and MEAM1 whitefly, Ghanim et al. (1998) showed TYLCV DNA could be detected in 81% of the eggs, 37% of the nymphs and 57% of the adults when they used more mature female adults (5–8 days after eclosion) to produce the eggs. Moreover, 10% of the progeny adults could infect tomato plants indicating transovarial transmission. Using two whitefly species, MEAM1 and MED, and two virus species, TYLCV and tomato yellow leaf curl Sardinia virus, Bosco et al. (2004) showed tomato yellow leaf curl Sardinia virus DNA could be detected in eggs and nymphs and to a lesser extent in adults of the first-generation progeny, while no TYLCV DNA was detected in eggs, nymphs and adults. However, the developmental stage of the whiteflies used to initiate infestation in these studies was not specified in their reports. Wang et al. (2010) studied vertical transmission of TYLCV and tomato yellow leaf curl China virus by MEAM1 and MED; they used newly emerged (0–24 h post emergence) whitefly adults to acquire virus and then oviposit on new plants, virus DNA was detected in eggs and nymphs but not in adults of the TYLCV-MEAM1 treatment. Again using newly emerged (0–8 h post emergence) whitefly adults for vertical transmission experiments Pan et al. (2012) obtained similar results; TYLCV DNA was detected in eggs and nymphs, but not in pupae and adults of the first generation progeny.

A recent study from our laboratory showed that both MEAM1 and MED can efficiently transmit TYLCV to their offspring via ova when more mature adults at 11 days after eclosion (DAE) were tested (Wei et al., 2017). Since transovarial transmission is reported to be a feature of circulative propagative plant virus (Mims, 1981), many studies have focused on TYLCV replication in its vector and this issue remains controversial. Sinisterra et al. (2005) identified the viral transcripts from both the viral and complementary strand by using real-time RT-PCR, suggested the existence of a double strand DNA replicative form in the vector. Further, Pakkianathan et al. (2015) showed that the virus amount of TYLCV in whitefly could increase for the first few days after virus acquisition. However, Becker et al. (2015) quantified the viral loads and complementary strands of both Mld and IL strains of TYLCV in whitefly, the results showed that the DNA of both strains remained stable after virus acquisition had stopped. Similarly, Sánchez-Campos et al. (2016) revealed that viral DNA quantities within whitefly body did not increase for time points up to four days after virus acquisition. Although TYLCV has been widespread in China for some years, little data are available on the vertical transmission capacity of this virus by indigenous whiteflies. Indigenous whiteflies are distributed in most regions of south China, and are able to cause serious damage to vegetables in some localities (Hu et al., 2011). As invasive and indigenous whiteflies are morphologically similar but genetically distinct, and invasive whiteflies can transmit TYLCV to its offspring with high efficiency, the transovarial transmission of TYLCV by indigenous whiteflies warrants investigation.

In this study, we characterized the transovarial transmission of TYLCV by seven species of the *Bemisia tabaci* complex indigenous to China. We found that TYLCV was able to cross the midgut and reach the haemolymph of whiteflies of all the seven species tested. While TYLCV could be transmitted by these whiteflies via ova to their eggs and nymphs, the virus was never detected in the ensuing adults, indicating that TYLCV is lost in the F1 offspring before they have developed to adults. Moreover, the capacity for transovarial transmission varies depending on the whitefly species.

## 2 Materials and methods

### 2.1 Virus, insects and plants

An infectious clone of the TYLCV isolate SH2 (GenBank accession No. AM282874.1) was provided by Professor Xueping Zhou, Institute of Biotechnology, Zhejiang University. Colonies of eight whitefly species (seven indigenous to China plus the invasive MEAM1) (Table 1) were maintained on cotton (*Gossypium hirsutum* cv. Zhe-Mian 1793) in insect proof cages in climate chambers at 26 ± 2 °C under 14 h photoperiod, 60 ± 10% relative humidity. The purity of each of the eight whitefly cultures was monitored every three to four generations by randomly sampling 20–40 adults using the mitochondrial cytochrome oxidase I (mt*COI*) PCR-restriction fragment length polymorphism and mt*COI* sequencing (Qin et al., 2013). To obtain TYLCV-infected tomato plants (*Solanum lycopersicum* Mill. cv. Hezuo 903), plants at 3–4 true-leaf stage were agro-inoculated with a TYLCV infectious clone (Li et al., 2019). Plants were grown to the 6–7 true-leaf stage before used in experiments. Infection of tomato plants was determined by both the inspection of disease symptoms and detection of viral DNA. All tomato and cotton plants were planted singly in 1.5 L pot with potting mix (peat moss, vermiculite, organic fertilizer, perlite in a 5:1:1:1 ratio by volume) and grown in insect proof greenhouses maintained at 25 ± 3 °C under a 14 h photoperiod from 6:00 to 20:00 (natural lighting supplemented with artificial lighting), 60 ± 10% relative humidity. Prior to an experiment, all leaves from each plant were checked to ensure they were insect-free.

**Table 1:** Eight species of the whitefly *Bemisia tabaci* complex collected from China and used in the experiments.

Whitefly species	Locality of collection	Source plants	mt*COI* GenBank accession no.
Asia 1	Honghe, Yunnan	Sweet potato	KC540757
Asia II 1	Jiande, Zhejiang	Cotton	DQ309077
Asia II 3	Yuhang, Zhejiang	Soybean	DQ309076
Asia II 6	Baise, Guangxi	Sweet potato	KC540758
Asia II 7	Guangzhou, Guangdong	Croton	EU192043
China 1	Yuhang, Zhejiang	Sweet potato	GQ303180
China 2	Guangzhou, Guangdong	Pumpkin	AY686072
MEAM1	Hangzhou, Zhejiang	Cabbage	KM821540

### 2.2 Acquisition of TYLCV by whiteflies

For each whitefly species, non-viruliferous adult whiteflies at 0–2 DAE or 7–9 DAE, were drawn from their respective culture and transferred to feed on a TYLCV-infected tomato plants enclosed in an insect-proof cage for 48 h or 168 h acquisition access period (AAP) as designated in each of the following experiments.

### 2.3 Transmission of TYLCV to tomato plants by whiteflies

For this experiment, only six indigenous whitefly species were used. The tests of Asia II 1 and MEAM1 had been performed previously (Li et al., 2010), we did not include them in this experiment. For each indigenous whitefly species, approximately 300 adults at 0–2 DAE were caged with leaves of TYLCV infected tomato plants for 48 h. The viruliferous female adults were then collected in groups of 10 each to feed on the second leaf from the bottom of an un-infected tomato plant at the three true-leaf stage using a leaf-clip cage. After a 48 h inoculation access period, all whiteflies were removed and the plants were then sprayed with imidacloprid at a concentration of 20 mg/L to kill any eggs laid on the leaves. The plants were kept for symptom development in insect-proof cages. For each whitefly species, we had fifteen plants. The tomato plants were examined 25–30 days thereafter to determine TYLCV infection first by symptoms visually and then examined by PCR. The top fully-expanded leaf of each tomato plants was collected, its DNA extracted with the Plant Genomic DNA Kit (TIANGEN, China) and used as template for PCR detection using various primers (Table 2).

**Table 2:** Primers used in this study.

Purpose	Primer	Sequence (5′-3′)	Reference
Virus detection by conventional PCR	TYLCV F	ATCGAAGCCCTGATATCCCCCGTGG	Ghanim et al. (2001)
	TYLCV R	CAGAGCAGTTGATCATG	
Virus quantification in whiteflies by qPCR	TYV1 RTF	GAAGCGACCAGGCGATATAA	Sinisterra et al. (2005)
	TYV1 RTR	GGAACATCAGGGCTTCGATA	
Normalizer in qPCR	Bt *β-Actin* RTF	TCTTCCAGCCATCCTTCTTG	Sinisterra et al. (2005)
	Bt*β-Actin* RTR	CGGTGATTTCCTTCTGCATT	

### 2.4 Transovarial transmission of TYLCV by whitefly

For each of the eight whitefly species, non-viruliferous adults at 7–9 DAE were transferred to feed on a TYLCV-infected plant enclosed in an insect-proof cage for 48 h AAP. Then the viruliferous adult whiteflies were collected in groups of 10 each (female/male = 1:1). For each replicate, a group of whiteflies was enclosed in a leaf-clip cage placed on the abaxial surface of a leaf of cotton plant, a non-host plant of TYLCV. The adults were left on the leaf to feed, mate and oviposit for 72 h. Then, all adults were removed and eight eggs were collected from each replicate using disposable sterilized needles (one needle for each individual) and stored at −20 °C for detection of virus DNA. The remaining eggs were left on the leaf to develop. After 7 and 21 days, eight 1st instar nymphs and eight adults were collected respectively from each replicate, using disposable sterilized needles (one needle for each individual) and stored at −20 °C for detection of virus DNA.

### 2.5 DNA extraction and PCR detection of TYLCV DNA in whitefly egg, nymph, haemolymph and whole body

The DNA from individual whitefly eggs, 1st instar nymphs and adults was extracted following the methods of Wei et al. (2017). Each whitefly egg, nymph and adult was individually placed on parafilm and ground in 10 μl for egg or nymph, or 30 μl for adult, of ice-cold lysis buffer (50 mmol/L KCl, 10 mmol/L Tris, 0.45% Tween 20, 0.2% gelatin, 0.45% Nonidet P-40, 60 mg/L proteinase K with pH at 8.4). Extracts were incubated at 65 °C for 1 h and 100 °C for 10 min, and then centrifuged for 10 s. The aqueous supernatant was used as template for PCR amplification. Samples of haemolymph from individual whiteflies were collected using the protocol described in Pan et al. (2018a). A single whitefly was placed into PBS on glass slides, and then the abdomen was cut to release the content, the midgut was removed, and the remaining liquid was collected. Then 10 μl of lysis buffer was added and the samples were incubated at 65 °C for 1 h and then 100 °C for 10 min to be used as template for PCR detection using various primers (Table 2).

### 2.6 Immunofluorescence Assay

Dissection of adult whiteflies from each of the seven indigenous species of whiteflies as well as MEAM1 was performed to examine the localization of TYLCV in the vector. In our preliminary experiments, we found that for the seven indigenous species of whiteflies, the immunofluorescence signals of the virus in midguts (MG) and primary salivary glands (PSG) were difficult to observe when the samples were collected after a 48 h AAP, but became observable after a 168 h AAP. Thus in the experiment here we collected whitefly samples of each of the eight species (Table 1) after a 168 h AAP on TYLCV-infected plants and then dissected the whiteflies to collect samples of MG and PSG for immunofluorescence assay.

In order to compare the efficiency of TYLCV penetration into the ovary of different putative whitefly species, whiteflies at 11 DAE were dissected for ovaries after a 48 h AAP on TYLCV-infected plants. The 48 h AAP was exercised here to parallel the AAP used in the transovarial transmission experiment. The specimens were fixed in 4% paraformaldehyde (MultiSciences Biotech., China) at room temperature for 1 h, and washed in TBS three times followed by 30 min in 0.1% Triton X-100, then washed in TBST three times. Specimens were then blocked in TBST containing 1% BSA for 2 h at room temperature. The anti-TYLCV coat protein monoclonal antibody (provided by Professor Xueping Zhou, Institute of Biotechnology, Zhejiang University), which has been used widely in the previous studies in our lab (Wei et al., 2014, Wei et al., 2017, Guo et al., 2018), was added at a dilution of 1:400 and the specimens were incubated overnight at 4 °C. After washing in TBST three times, specimens were incubated with goat anti-mouse secondary antibody labeled with Dylight 488 or Dylight 549 with a dilution of 1:400 in TBST for 2 h at room temperature. Finally, after washing in TBST three times, the nucleus of specimens were stained with 100 nM DAPI (4′,6-diamidino-2-phenylindole, Abcam, USA) at room temperature for 5 min. All samples were examined using a Zeiss LSM710 confocal microscope (Zeiss, Germany).

### 2.7 Analysis of virus quantity in whitefly whole body and offspring

For each whitefly species, approximately 150 non-viruliferous adult whiteflies at 7–9 DAE were transferred to feed on a TYLCV-infected tomato plant enclosed in an insect-proof cage for 48 h AAP. For analysis of quantity of the viral DNA in whitefly whole body, female adults were collected in groups of 15 for each replicate into 80 μl ice-cold lysis buffer. Extracts were incubated at 65 °C for 1 h and 100 °C for 10 min, and then centrifuged. The aqueous supernatant was used as a template for subsequent real time PCR analysis (qPCR). Due to an accident that happed to the adult sample of Asia II 3, we missed the opportunity to examine the quantity of virus in the adults of this species. For analysis of quantity of viral DNA in whitefly offspring, the eggs, 1st instar nymphs and adults examined in the transovarial transmission bioassay (described above) were collected and examined individually using qPCR, and the offspring at different developmental stages of nonviruliferous whiteflies were analyzed as a negative control. qPCR were performed using a CFX96™ Real-Time PCR Detection System (Bio-Rad, USA) with SYBR Premix Ex Taq II (Takara, Japan) and the primers used in the qPCR are listed in Table 2. The relative abundance of viral DNA was calculated using 2^-ΔCt^ method as normalized to the level of whitefly *β*-actin gene (Sinisterra et al., 2005).

### 2.8 Statistical analysis

The relative abundance of TYLCV DNA in female adults of all whitefly species was compared by nonparametric Mann-Whitney test at a 0.05 significance level. All data are presented as the mean ± standard error of the mean (mean ± SEM). All the data analysis was performed using IBM Statistics SPSS 20.0 and EXCEL.

## 3 Results

### 3.1 All the tested whitefly species can transmit TYLCV

**Fig. 1:**
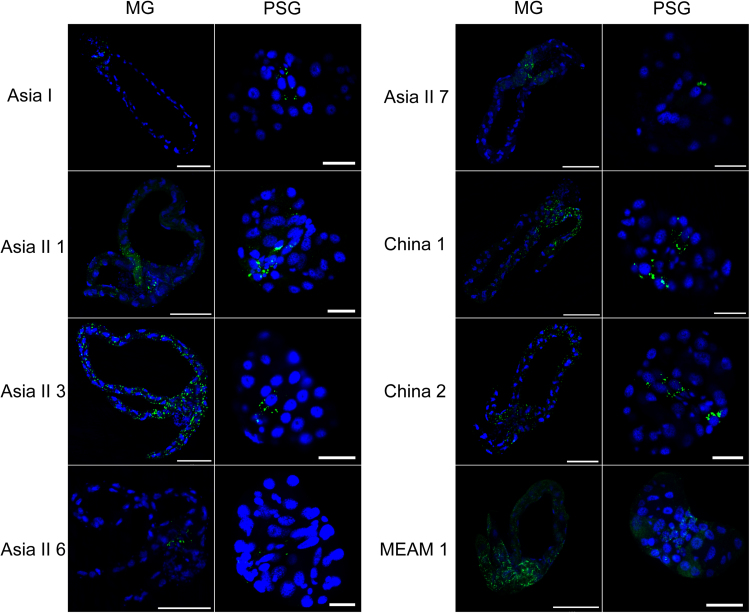
Localization of TYLCV in whitefly midgut and primary salivary glands. TYLCV localization in midguts and primary salivary glands dissected from different species of whiteflies that had fed on TYLCV-infected tomato leaves for 168 h. TYLCV was detected by a mouse anti-TYLCV antibody and a goat anti-mouse secondary antibody conjugated to FITC (green); nuclei were stained with DAPI (blue). Left panels show the midgut (MG) (Scale bar, 100 µm) and right panels show the primary salivary gland (Scale bar, 20 µm).

Using PCR, TYLCV DNA was detected in the haemolymph of each of the six indigenous species after they had fed on virus-infected tomato plants for a 48 h AAP (Table 3). Immunofluorescence detected virus signals in midgut and primary salivary glands of each of the seven indigenous whitefly species as well as MEAM1 serving as a positive control (Fig. 1). All the six whitefly species tested in this study were able to transmit TYLCV to uninfected tomato plants with various levels of efficiency (Table 3).

**Table 3:** PCR detection of tomato yellow leaf curl virus DNA in the haemolymph and the TYLCV transmission efficiency of different species of whitefly.

Whitefly species	Source	Viral DNA	
		Haemolymph	Plant
Asia 1	This study	86.7% (26/30)	40.0% (6/15)
Asia II 3	This study	80.0% (24/30)	73.3% (11/15)
Asia II 6	This study	46.7% (14/30)	40.0% (6/15)
Asia II 7	This study	60.0% (18/30)	73.3% (11/15)
China 1	This study	100% (30/30)	73.3% (11/15)
China 2	This study	96.7% (29/30)	93.3% (14/15)
Asia II 1	(Li et al., 2010)	+^a^	+
MEAM1	(Wei et al., 2017)	+	+

a The symbol “+” indicates detection of TYLCV DNA using PCR.

### 3.2 Transovarial transmission efficiency of TYLCV by different whitefly species

**Fig. 2:**
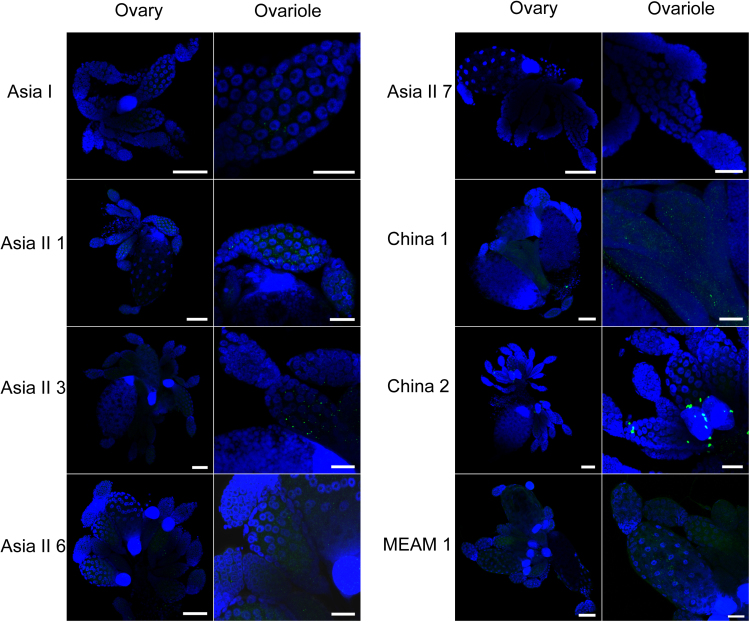
Localization of TYLCV in whitefly ovary. TYLCV localization in ovaries dissected from whiteflies of different species at 11 DAE that had fed on TYLCV-infected tomato leaves for 48 h. TYLCV was detected by a mouse anti-TYLCV antibody and a goat anti-mouse secondary antibody conjugated to 549 (green); nuclei were stained with DAPI (blue). Left panels show a whole view of ovary (Scale bar, 50 µm) and right panels show the enlarged view of ovarioles (Scale bar, 20 µm).

Immunofluorescence assays were conducted to detect TYLCV signals in ovaries and ovarioles in each of the seven indigenous whitefly species as well as MEAM1, which served as a positive control. Virus signals were easily observed in the ovarioles of Asia II 1, Asia II 3, China 1 and China 2, and weak signals were seen in the ovaries of Asia 1 and Asia II 6, but no signal was detected in the ovaries of Asia II 7 (Fig. 2). Next, we examined for the presence of TYLCV DNA in the offspring of different stages via transovarial transmission by each of the seven indigenous species of whiteflies as well as MEAM1. TYLCV DNA was detected in 1–5% of the eggs of Asia 1 and Asia II 6, and in 30–68% of the eggs of Asia II 1, Asia II 3, China 1, and China 2, respectively; however, no signal of TYLCV DNA was detected in the eggs of Asia II 7. Subsequently, TYLCV DNA was detected in 50-56% of the 1st instar nymphs of Asia II 3 and China 1, and in 2.5% of the 1st instar nymphs of Asia 1, but not in the other four species of whiteflies. TYLCV DNA was not detected in any of the F1 adults of all the seven indigenous species of whiteflies (Table 4).

**Table 4:** Frequency of transovarial transmission of TYLCV by different species of whiteflies in the *Bemisia tabaci* species complex.

Whitefly species	Whitefly age, DAE	Source of data	Viral DNA detected in different life stages		
			Egg	Nymph	Adult
Asia 1	9–11	This study	1.3% (1/80)	2.5% (2/80)	(0/80)
Asia II 1	9–11	This study	31.3% (25/80)	(0/80)	(0/80)
Asia II 3	9–11	This study	61.3% (49/80)	56.3% (45/80)	(0/80)
Asia II 6	9–11	This study	5.0% (4/80)	(0/80)	(0/80)
Asia II 7	9–11	This study	(0/80)	(0/80)	(0/80)
China 1	9–11	This study	68.8% (55/80)	50.0% (40/80)	(0/80)
China 2	9–11	This study	47.5% (38/80)	(0/80)	(0/80)
MEAM1	9–11	This study	62.5% (25/40)	67.5% (27/40)	75.0% (30/40)
MEAM1	11	(Wei et al., 2017)	91.7% (55/60)	68.3% (41/60)	76.7% (46/60)

Viral DNA was not detected in any of the individuals.

### 3.3 TYLCV acquisition by different species of whiteflies

**Fig. 3:**
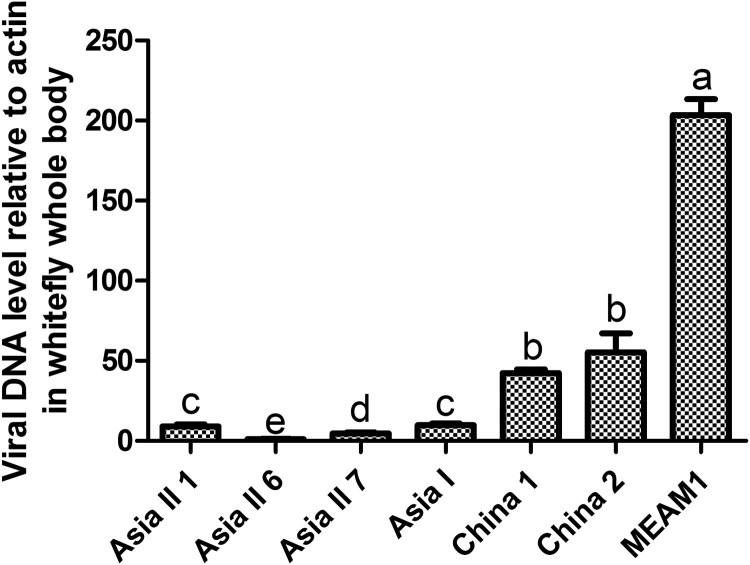
Quantity of TYLCV in different species of *B. tabaci* complex. Quantity of virus in the whole body of whiteflies of different species that had fed on TYLCV-infected tomato leaves. Whiteflies were collected at 7–9 DAE, and then they were provided a 48 h AAP on TYLCV-infected tomato leaves. After virus acquisition, the quantity of TYLCV in the whitefly whole body was analyzed using qPCR. The values represent mean ± SEM of four replicates for each whitefly species. Different letters above the bars indicate significant differences (nonparametric Mann-Whitney test, *P* < 0.05).

In order to examine any connection between virus acquisition capacity and virus vertical transmission efficiency in the different species of whiteflies, the virus in the whole body of different whitefly species was quantified using qPCR after they had fed on TYLCV-infected tomato for 48 h. The virus quantity was highest in MEAM1, followed by China 2, China 1, and finally by Asia I, Asia II 1, Asia II 7, and Asia II 6 (Fig. 3). The variation in the quantity of virus acquired by different species of whiteflies largely agrees with that of transovarial transmission efficiency by these species (Table 3).

### 3.4 TYLCV quantity in offspring of different developmental stages of two species of whiteflies

**Fig. 4:**
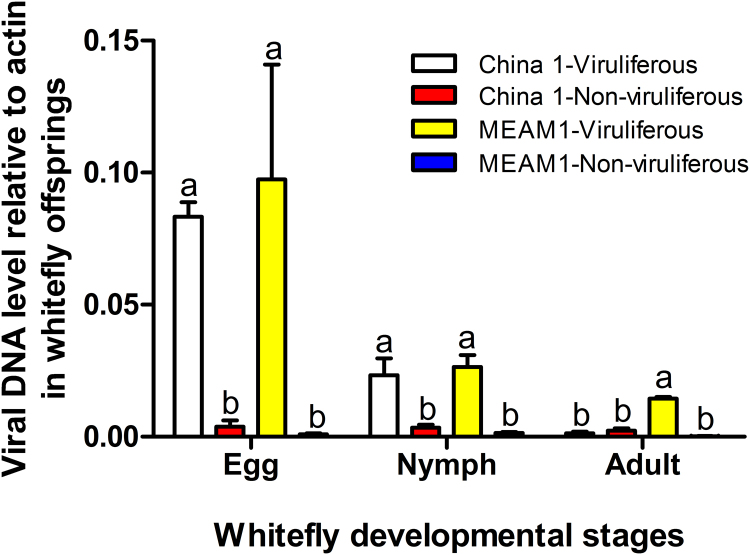
Relative quantity of virus in the offspring of viruliferous whiteflies of different species of the *B. tabaci* complex. The values represent mean ± SEM of 3–5 replicates. Different letters above the four columns of a given developmental stage indicate significant differences (nonparametric Mann-Whitney test, *P* < 0.05).

In order to examine whether the quantity of virus in nymphs of the offspring of viruliferous whiteflies was a major determinant of the success of transovarial transmission, we quantified the virus in two species, China 1 and MEAM1, that had similar proportions of TYLCV infection at the egg and 1st instar nymph stages after transovarial transmission, but displayed complete disparity in the proportions of the adults harbouring the virus (Table 4). The quantities of virus in the egg and 1st instar nymph did not differ significantly between China 1 and MEAM1. However, when the nymphs have developed to adulthood, no virus was detected in China 1 whereas virus was readily detected in MEAM1 (Fig. 4).

## 4 Discussion

TYLCV was serologically detected in the haemolymph of the six indigenous species (Table 3) and in the primary salivary glands of the eight species we tested (Fig. 1). In addition, we found that viruliferous adult parent whiteflies of all six species we tested could transmit TYLCV to un-infected tomato plants, indicating that TYLCV could circulate and cross the salivary gland escape barrier as well (Table 3). Immunofluorescence assays of whitefly ovaries indicated that TYLCV could cross the transovarial transmission barrier of all species of whitefly except Asia II 7 (Fig. 2). TYLCV DNA could not be detected in the eggs, nymphs or adults of the offspring produced by viruliferous adults of Asia II 7. For the other whitefly species, TYLCV DNA could be detected in the offspring eggs to varying degree, and in approximately 50% of the offspring nymphs of Asia II 3 and China 1, but not the nymphs of the remaining species (Table 4). The differences in the capacity of TYLCV transovarial transmission among the whitefly species seemed to be positively associated with the quantity of virus in their body (Fig. 3). In addition, the quantity of virus in offspring eggs or nymphs may not be a good indicator for the success of trans-generation transmission of TYLCV via the transovarial mode in whiteflies (Fig. 4). Altogether, these findings indicate that the seven species indigenous to China and MEAM1 vary in their capacity of transovarial transmission of TYLCV, from complete absence, to reaching the eggs, or to the nymph stage, or to the adult. However, none of the seven indigenous species of whiteflies could transmit the virus to the adults of the next generation via transovarial transmission.

Previous studies have shown that the whitefly species of MEAM1 and MED could transmit TYLCV to its progeny adults, and these progeny adults could transmit the virus to uninfected host plants. Moreover, the second-generation offspring of viruliferous whiteflies were able to inoculate tomato plants with efficiency similar to that of the first-generation (Wei et al., 2017, Ghanim et al., 1998). In addition, Wei et al. (2017) found that whitefly vitellogenin (Vg) acts as an essential player in the process of transovarial transmission. By means of interacting with the vector’s Vg, TYLCV virions could enter the oocytes of MEAM1 and then be carried over to the next generation. In contrast, the coat protein of papaya leaf curl China virus does not interact with the MEAM1′s Vg, and this virus is not transovarially transmitted (Wei et al., 2017). It is likely that the Vg of Asia II 7 does not interact with TYLCV coat protein, and consequently the virus is not transovarially transmitted.

The seven species of whiteflies indigenous to China and MEAM1 used in the experiments here were examined previously in this laboratory for their endosymbionts, and the data show that they vary in the community of endosymbionts (Bing et al., 2013, Zhang, 2016). One potential factor contributing to the between-species variation of transovarial transmission might be the differences of endosymbionts between the whitefly species. The role of endosymbionts of whiteflies in their viral transmission has attracted much attention, but very few attempts have been made to examine the role experimentally. This situation has occurred largely due to the extremely poor feasibility of working with whitefly endosymbionts, which cannot be cultured in vitro, and each of them does not respond specifically to antibiotic treatments. Consequently, it is hardly feasible to establish whitefly genetic lines, which share the same genetic background but differ only in the lack or bearing of a given endosymbiont, for experimentation (Zhang et al., 2015, Shan et al., 2016). Gottlieb et al. (2010) reported, probably for the first time, that the endosymbiont *Hamiltonella* residing in MEAM1 facilitates the host’s capacity of TYLCV transmission. And four years later, the same research group reported that the endosymbiont *Rickettsia* residing in MEAM1 likewise promotes the host’s capacity of TYLCV transmission (Kilot et al., 2014). The experiments in these two studies were meticulously conducted, but the results have been often questioned largely because of the whitefly lines used in the experiments were derived from different isofemales and their identity of genetic background cannot be strictly ensured. The findings reported from some other studies on this topic, such as Su et al. (2013) and Xue et al. (2012), are apparently problematic, because the whitefly lines used in the experiments, which were assumed to be free of a given endosymbiont such as *Hamiltonella*, were later found harbouring that endosymbiont (Zhang et al., 2015). With the current status of knowledge, it is risky to speculate any effects of the endosymbionts on the host’s capacity of TYLCV transmission.

When the quantities of TYLCV were compared in different developmental stages of the offspring produced by viruliferous MEAM1 and China 1, no significant differences were found between the two species of whitefly vectors. However, while MEAM1 could carry the virus to the adult stage, China 1 was unable to do so. The whitefly life cycle consists of egg, nymph and adult, and the transitions between stages are achieved through hatching and eclosion. These remoulding transitions between stages would not happen without dramatic metamorphosis, which is likely to play a significant role in transovarial transmission of a virus. Thus, unravelling the molecular mechanisms underlying the retention of the virus from one stage to the next of the vector may shed light on the process of transovarial transmission of virus or even vector’s transmission of virus in general.

In this study, seven whitefly species indigenous to China were compared for their efficiency in transovarial transmission of TYLCV. In view of the efficient transovarial transmission of TYLCV demonstrated previously by MEAM1 and MED (Ghanim et al., 1998, Wei et al., 2017), the indigenous species examined in this study exhibit lack or only half success in transovarial transmission. Tomato yellow leaf curl disease was initially recognized in the Middle East in the 1930s and then TYLCV was identified in the early 1960s (Cohen and Nitzany, 1966). The divergence of the species MEAM1 and MED from the other whitefly members was estimated to occur 12 million years ago in their Middle East-Asia Minor and Mediterranean origin (Boykin et al., 2013). As TYLCV was detected in China only about 15 years ago (Wu et al., 2006), the whiteflies indigenous to China have been associated with TYLCV only for a short period of time. Therefore, MEAM1 and MED may have been associated with TYLCV for much longer time than the whiteflies indigenous to China. Long-term association of MEAM1 and MED with TYLCV may assist in the development of compatibility between vectors and viruses including transovarial transmission.

In conclusion, the current study indicates that the seven species of whiteflies indigenous to China show apparent variations in transovarial transmission of TYLCV, from none to being able to reach the nymphal stages of the vectors, but none of them can transmit the virus to the adult stage of their progeny. The findings of the variation in efficiency of transovarial transmission of TYLCV between different whitefly species of the *B. tabaci* complex not only provide valuable information to the development of management strategies against these pests, but also call for investigations on the molecular mechanisms underlying the various changes in transovarial transmission of the virus by different whitefly vectors.
